# Assessing Pharmacodynamic Effects of Antiplatelet Agents With Different Mechanisms of Action

**DOI:** 10.1161/JAHA.120.020859

**Published:** 2021-04-21

**Authors:** David J. Schneider, Heidi S. Taatjes‐Sommer, Jayne Prats

**Affiliations:** ^1^ Cardiovascular Division Departmnt of Medicine and Cardiovascular Research Institute University of Vermont Burlington VT; ^2^ ELYSIS LLC Carlisle MA

**Keywords:** Platelets

The FABOLUS‐FASTER (Facilitation Through Aggrastat or Cangrelor Bolus and Infusion Over Prasugrel: A Multicenter Randomized Open‐Label Trial in Patients With ST‐Elevation Myocardial Infarction Referred for Primary Percutaneous Intervention) trial compared pharmacodynamic effects of different antiplatelet agents in patients with ST‐segment–elevation myocardial infarction.^1^ The inhibitory effects of cangrelor assessed by light transmission aggregometry (LTA) were substantially lower than previously reported.^2^ This study was designed to assess methodologic issues potentially contributing to the differences observed by using similar experimental conditions, albeit with the in vitro addition of antiplatelet agents.

Activation of platelets by ADP is mediated by both the P2Y_1_ and P2Y_12_ receptors.^3^ ADP induces a weak, transition activation by binding to the P2Y_1_ receptor and completes activation by binding to the P2Y_12_ receptor. A P2Y_12_ antagonist would not be expected to prevent transient aggregation induced by P2Y_1._ Thus, previous pharmacodynamic studies^2^ have quantified final rather than maximal aggregation. Glycoprotein IIb/IIIa inhibitors would be expected to exert similar effects on final and maximal aggregation. Both cangrelor and tirofiban bind reversibly. Tirofiban is cleared by renal excretion, whereas cangrelor is metabolized (t_1/2_ 3–6 minutes). The reversible binding and metabolism of cangrelor raises the possibility that delay in performance of assays may reduce the pharmacodynamic effect.

To assess the impact of comparing maximal with final aggregation, and delay in processing of samples on LTA, blood from 10 healthy individuals was anticoagulated with citrate and spiked with pharmacologic concentrations of cangrelor. The study was approved by the institutional review committee and all subjects gave informed consent. Data that support the findings are available from the corresponding author upon reasonable request. Preliminary experiments identified concentrations of cangrelor that would achieve results seen in patients^2^ with residual aggregation of <20%. Aliquots of blood were spiked with 86 ng/mL (n=6), 215 ng/mL (n=10), and 430 ng/mL (n=4). Similarly, citrate anticoagulated blood from 5 healthy individuals was spiked with pharmacologic concentrations of tirofiban (30 and 50 ng/mL). Aggregation induced by 20 micromolar ADP in platelet‐rich plasma was assessed within 30 minutes (time 0) and after 1, 2, 4, and 6 hours. Differences were assessed with a repeated measure ANOVA and pairwise comparisons with time 0 were assessed by Bonferroni paired *t* test.

Both maximal and final (after 6 minutes) aggregation decreased when performance of LTA was delayed (maximal and final aggregation were comparable in the absence of added antiplatelet agent, mean±SD; final aggregation at time 0: 71±16, 1 hour: 56±16, 2 hours: 57±14, 4 hours: 49±16, and 6 hours: 53±14 [*P*<0.01]). Final but not maximal aggregation increased modestly when LTA was performed after 1 and 2 hours with blood spiked with cangrelor (cangrelor 86 ng/mL, time 0: 16±14, 1 hour: 22±15, and 2 hours: 23±13 [*P*<0.02]).

No significant difference was seen in the inhibition of maximal and final aggregation with tirofiban (Figure). Final aggregation was inhibited to a greater extent (*P*<0.01) than maximal aggregation (Figure) in blood spiked with cangrelor.

In summary, while results should be confirmed in patients treated with antiplatelet agents, our results demonstrate that delay in performance of LTA influences results. Importantly, while the glycoprotein IIb/IIIa inhibitor tirofiban inhibits maximal and final aggregation to a similar extent, significant differences were apparent when cangrelor was assessed with maximal compared with final aggregation. This difference is likely to be accentuated by the increased platelet reactivity associated with acute myocardial infarction.^4^ Maximal aggregation quantified by LTA in blood from cangrelor‐treated patients reflects platelet activation induced by P2Y_1_ and is, therefore, not a reflection of the antiplatelet effects of cangrelor.

Our findings are consistent with the multiple electrode (impedance) aggregometry results of FABOLUS‐FASTER.^1^ Greater inhibition was apparent with cangrelor and differences between cangrelor and tirofiban were less.

The methodologic issues identified here underscore the challenges associated with predicting clinical outcomes with pharmacodynamic studies and support a standardized approach to pharmacodynamic assessment. In addition, they underscore the premise that results from pharmacodynamic studies should not outweigh established clinical efficacy. Tirofiban inhibits aggregation of platelets; however, a glycoprotein IIb/IIIa inhibitor does not inhibit other aspects of platelet activation such as platelet‐leukocyte aggregation, release of granular products, and the promotion of thrombin generation. Cangrelor inhibits activation of platelets mediated by P2Y_12_ that limits amplification by agonists such as thrombin and collagen.^5^ The clinical implications of the differing pharmacodynamic effects have not been defined. A randomized clinical trial appropriately sized to compare clinical events is required to determine whether 1 agent is superior in the prevention of clinical events.

## Sources of Funding

This work was supported by a grant from Chiesi.

## Disclosures

Schneider was the principal investigator on a grant supporting this study. Jayne Prats is a consultant for Chiesi USA, the current owner of Kengreal (cangrelor). Heidi Taatjes‐Sommer has no disclosures to report.

**Figure 1:**
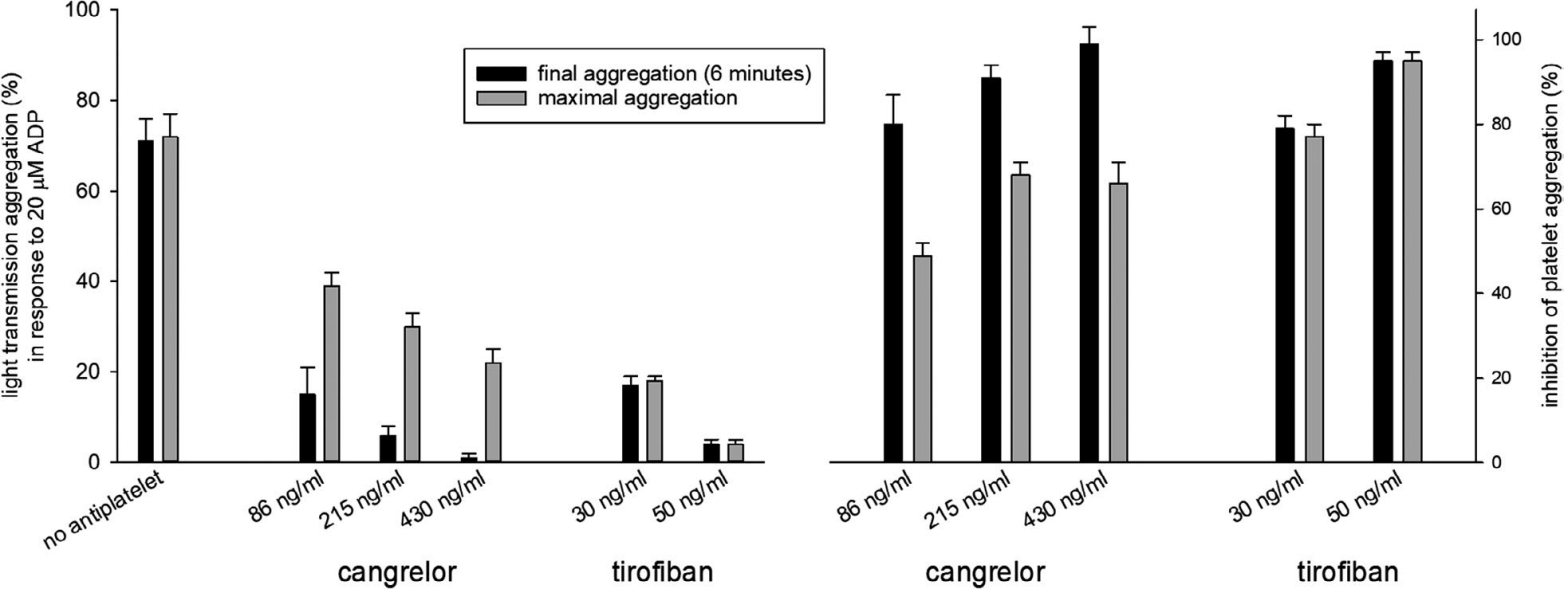
Aggregometry was performed with pharmacologic concentrations of cangrelor and tirofiban within 30 minutes. In blood spiked with cangrelor maximal aggregation occurred early after addition of ADP and disaggregation followed. With each concentration of cangrelor (mean±SEM), final (after 6 minutes) was less (*P*<0.01) than maximal aggregation. Inhibition of aggregation is shown on the right. ADP indicates adenosine diphosphate.
